# The Genus *Enterococcus*: Between Probiotic Potential and Safety Concerns—An Update

**DOI:** 10.3389/fmicb.2018.01791

**Published:** 2018-08-03

**Authors:** Hasna Hanchi, Walid Mottawea, Khaled Sebei, Riadh Hammami

**Affiliations:** ^1^Nutraceuticals and Functional Proteomics Potential of Biodiversity in Tunisia, Higher Institute of Applied Biological Sciences of Tunis (ISSBAT), University of Tunis El Manar, Tunis, Tunisia; ^2^Faculty of Health Sciences, School of Nutrition Sciences, University of Ottawa, Ottawa, ON, Canada; ^3^Department of Microbiology and Immunology, Faculty of Pharmacy, Mansoura University, Mansoura, Egypt

**Keywords:** *Enterococcus*, probiotics, bacteriocin, health promotion, food safety, lactic acid bacteria, legislation

## Abstract

A considerable number of strains belonging to different species of *Enterococcus* are highly competitive due to their resistance to wide range of pH and temperature. Their competitiveness is also owed to their ability to produce bacteriocins recognized for their wide-range effectiveness on pathogenic and spoilage bacteria. Enterococcal bacteriocins have attracted great research interest as natural antimicrobial agents in the food industry, and as a potential drug candidate for replacing antibiotics in order to treat multiple drugs resistance pathogens. However, the prevalence of virulence factors and antibiotic-resistance genes and the ability to cause disease could compromise their application in food, human and animal health. From the current regulatory point of view, the genus *Enterococcus* is neither recommended for the QPS list nor have GRAS status. Although recent advances in molecular biology and the recommended methods for the safety evaluation of *Enterococcus* strains allowed the distinction between commensal and clinical clades, development of highly adapted methods and legislations are still required. In the present review, we evaluate some aspects of *Enterococcus* spp. related to their probiotic properties and safety concerns as well as the current and potential application in food systems and treatment of infections. The regulatory status of commensal *Enterococcus* candidates for food, feed, probiotic use, and recommended methods to assess and ensure their safety are also discussed.

## Introduction

Enterococci are lactic acid bacteria (LAB) comprising both pathogenic and commensal microorganisms ubiquitous in environment even as gut symbionts. Due to their tolerance to salts and acids, the strains of *Enterococcus* spp. are highly adapted to several food systems, they are also involved in the fermentation activity of traditionally manufactured cheese and dry sausages, in which it is believed that they contribute to the development of organoleptic characteristics of these products (Foulquié Moreno et al., 2006). In addition, several number of *Enterococcus* strains have been reported to produce antimicrobial compounds including bacteriocins. Bacteriocin production have be applied to preservation of a wide range of food products and is now being considered as a probiotic trait (Yang et al., 2014). besides, bacteriocins are considered as promising alternative to fight emerging antimicrobial resistance (Cotter et al., 2013; Hammami et al., 2013). Although certain antibiotic-resistant, infectious strains of enterococci, including *E. faecium*, have been identified in hospital patients, they very rarely present a risk of infection for humans outside healthcare settings (Sanders et al., 2010). To date, the genus *Enterococcus* has not yet obtained the status generally recognized as safe (GRAS) (Huys et al., 2013), but some members are used as probiotics and in production of feed additives to prevent diarrhea or to improve growth in animals (Franz et al., 2011). This situation has created a requirement for new regulation of probiotics in order to distinguish between safe and potentially harmful strains. In this review, probiotic potential, safety use as well as recent advances in knowledge about bacteriocin production by enterococci will be discussed, with emphasis on the potential opportunities for application in various fields.

## Legislation

**Figure 1:**
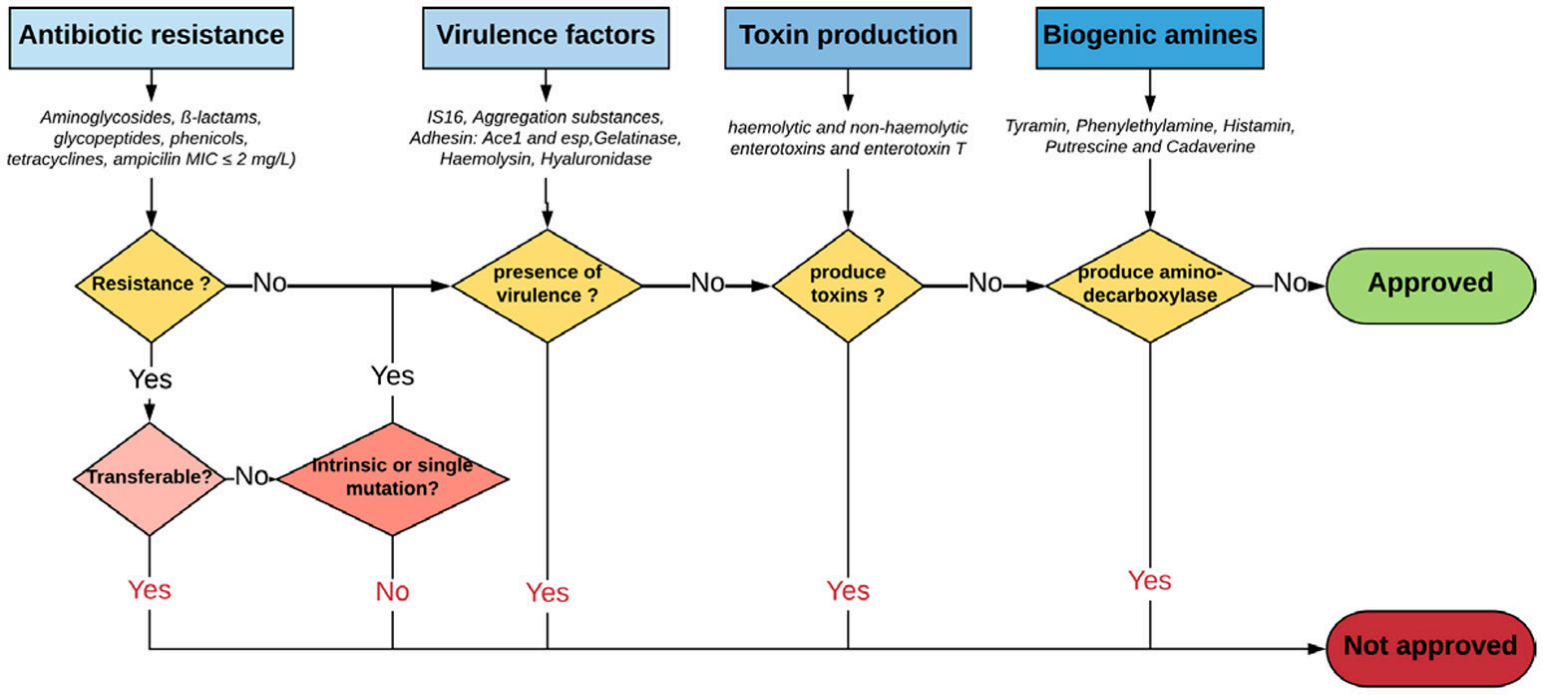
Proposed decision scheme for the safety assessment of *Enterococci* probiotic candidates leading to food/feed applications. Adapted from EFSA Panel on Biological Hazards (2011), EFSA (2012a) and Laulund et al. (2017).

With regard to safety and according to the Qualified Presumption of Safety (QPS) list from the European Food Safety Authority (EFSA) (https://www.efsa.europa.eu/en/topics/topic/qps), *Enterococcus* species are neither recommended for the QPS list (EFSA Panel on Biological Hazards et al., 2017) nor have GRAS status (Ogier and Serror, 2008), in spite of recent scientific knowledge allowing differentiation of commensal from pathogenic strains (Montealegre et al., 2016; Bonacina et al., 2017; Jung et al., 2017). In this regard, recent advances in molecular epidemiology based on molecular fingerprinting, multi-locus sequence typing, phenotypic studies and whole-genome analyses have provided further evidences that nosocomial strains of *Enterococcus* are genotypically different from commensal strains. For instance, *E. faecium* has been subtyped into three different clades: the hospital-associated clade A1, rarely found in healthy individuals; the animal-associated clade A2; and the community-associated clade B, commonly found in healthy individuals and rarely causes infections (Montealegre et al., 2016). Beukers and coauthors have compared complete genomes of *E. faecium* from the NCBI database to demonstrate differential clustering of commensal and clinical isolates, suggesting that these strains may be specifically adapted to their respective environments (Beukers et al., 2017). Similarly, the difference between ability of pathogenic and commensal *E. cecorum* isolates from different animal species to metabolize mannitol may be explained by a separate evolution of pathogenic *E. cecorum* isolates (Jung et al., 2017). In addition, investigation of the genome sequences of 4 groups of *Enterococcus* species from food origin including dairy, meat, probiotics and probiotics from dairy origin showed no correlation between isolation source/probiotic properties and phylogenetic signal neither at species or strain levels (Bonacina et al., 2017). Although further evidences are required, the advances outlined above support the call for new recommendations about probiotic legislative framework in order to distinguish between safe and potentially harmful *Enterococcus* strains. Yet, organizations such as the EFSA, the Advisory Committee on Novel Foods and Processes, ACNFP, and the Food Standards Agency permitted the use of certain strains of enterococci as a food additive and supplements based on a careful case-by-case assessment. In this case, the individual strain must be considered and health risks must be excluded for this specific strain (ACNFP, 1996; Franz et al., 2011; Lauková, 2011; EFSA, 2012a). The EFSA guidance (EFSA, 2012a) provides a methodology for distinguishing between safe and potentially harmful strains of *E. faecium* in animal nutrition. It is intended for use as feed additive producers submitting applications to EFSA for safety assessment. According to this guidance, strains to be used in animal nutrition shall be susceptible to ampicillin (MIC ≤ 2 mg/L) and shall not harbor one of the genetic elements *IS*16, *hylEfm*, and *esp*. For the evaluation of new probiotic candidates by EFSA, the full strain genome should be available (Brodmann et al., 2017). Specific strains of *E. faecium* and *E. faecalis* are the only enterococci used as probiotics or feed additives (Franz et al., 2011). The use of other enterococci species is subject to little or no regulation in spite of the increasing number of studies that elucidate the probiotic potential of some species such as *E. munditii, E. durans* and *E. hirae* (Nami et al., 2014; Pieniz et al., 2014; Gupta and Tiwari, 2015; van Zyl et al., 2016). Figure 1 illustrates a proposed decision scheme based on EFSA regulations for the safety assessment of enterococci probiotic candidates leading to food/feed applications.

**Graphical Abstract:**
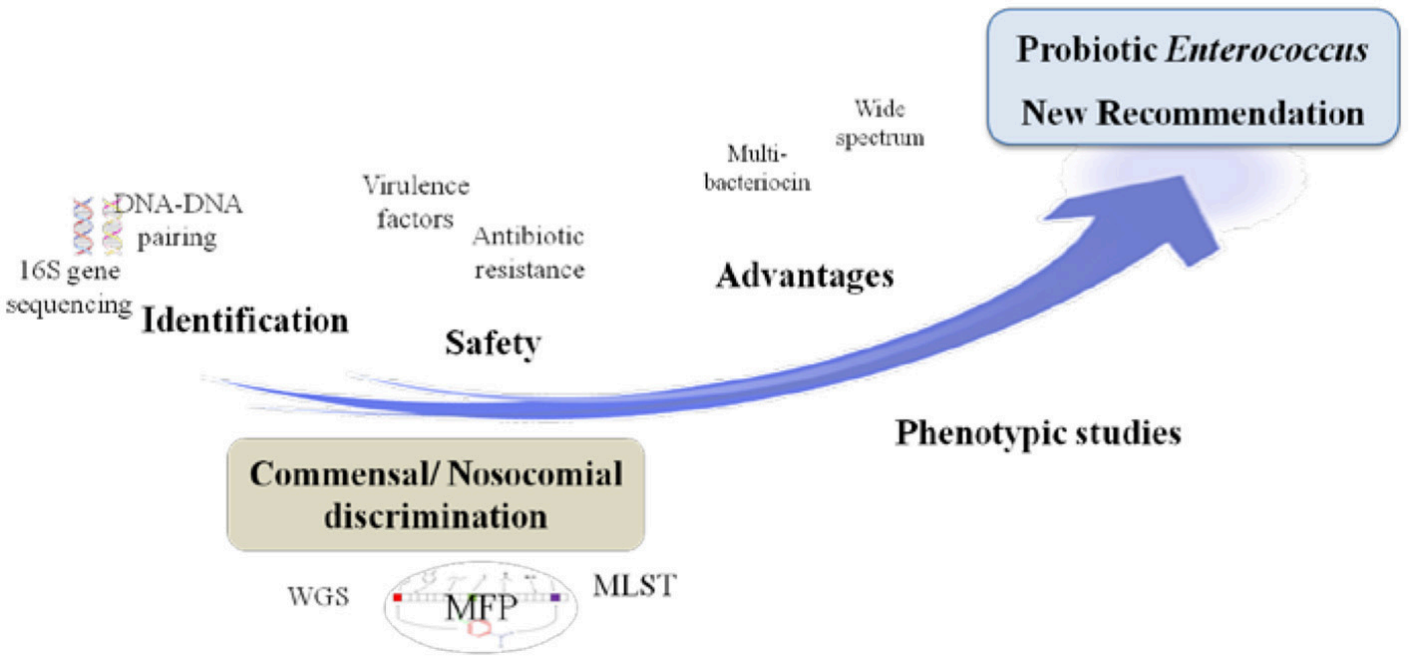


Bacteriocins are antimicrobial compounds produced by bacteria that have high potential of application as natural food additives to enhance food safety (Cotter et al., 2005; Yang et al., 2014; Egan et al., 2016). From the perspective of the current legislation, nisin and pediocin PA1/AcH has been the only bacteriocins approved for utilization as food additives by FDA in USA and EU regardless the considerable number of experimental work on the application of bacteriocins in food (Vignolo et al., 2012; Barbosa et al., 2017). With the emergence and rapid spread of resistance to conventional antibiotics, bacteriocins are considered to be an attractive alternative in the treatment of antibiotic-resistant bacterial infections (Cotter et al., 2013; Hammami et al., 2013), and a few are being tested in clinical trials (Behrens et al., 2017). Bacteriocins are potent, lowly toxic and target a narrow range of bacteria so they can act without affecting much of the natural microbiota which is a common drawback of conventional antibiotic use (Lohans and Vederas, 2012; Ołdak and Zielinska, 2017). The gene-encoded nature of bacteriocins makes them easily amenable through bioengineering to either increase their activity or specify target microorganism (Yang et al., 2014). Nevertheless, probiotic strains or bacteriocins intended for use as a therapeutic must undergo the regulatory process as a new drug, and must be authorized by the FDA (Venugopalan et al., 2010).

## Advantages of enterococci and their bacteriocins

Enterococci are extensively studied as potential candidate probiotics. Considerations for strain selection include several criteria such as molecular identification using genetic typing techniques, safety, capacity to survive intestinal transit, manufacturing, distribution, and targeted application. The functional requirements of probiotics include tolerance to human gastric juice and bile, adherence to epithelial surfaces, persistence in the human gastrointestinal tract (GIT), immune stimulation, antagonistic activity toward intestinal pathogens (such as *Helicobacter pylori, Salmonella* spp., *Listeria monocytogenes*, and *Clostridium difficile*), and the capacity to stabilize and modulate the intestinal microbiota. Bacteriocin production is now one of the desirable traits in the selection of a probiotic strain (Dobson et al., 2012). Strains belonging to the genus *Enterococcus* produce a wide variety of bacteriocins often called enterocins. They have been widely studied, mainly because they are active against Gram-positive foodborne pathogens, such as *L. monocytogenes* (Izquierdo et al., 2009). *E. faecium* and *E. faecalis* are the main producers of enterocins and to a lesser extent *E. mundtii, E. avium, E. hirae*, and *E. durans*. Some of these bacteriocins can be grouped with typical bacteriocins produced by LABs according to traditional classification (Cotter et al., 2005), whereas others could not be included. Franz et al. (2007) suggested grouping enterocins into a new four classes. Class I, lantibiotic enterocins, rarely found in enterococci are represented only by cytolysin (Christopher et al., 2005) and enterocin W (Sawa et al., 2012) both from *E. faecalis* isolates. The enterocin of this class is a two-component bacteriocin consisting of two linear peptides structurally different from other linear lantibiotics such as nisin A and Z as well as from smaller globular peptide lantibiotics. It contains lanthionine residues, which suggests their consideration as two-component lantibiotics. Class II, enterocins of the pediocin family. The Class II.1 of pediocin-like bacteriocins is divided into two subgroups according to sequence similarities (Lauková, 2011). Subgroup 1 includes enterocin A (Aymerich et al., 2000), mundticin (produced by *E. mundtii*) (Kawamoto et al., 2002) and enterocin CRL5 (Saavedra et al., 2004); and subgroup 2 includes enterocin P (Cintas et al., 1997) and enterocin M (Marekova et al., 2007), a variant of enterocin P. The Class II.2 refers to enterocins synthesized without a leader peptide such as two peptide bacteriocin L50 (A, B) (Cintas et al., 2000), enterocin Q (Cintas et al., 2000) and enterocin C (Maldonado-Barragan et al., 2009). Liu et al. (2011) reported that leaderless bacteriocins might possess a formyl group in their N-terminal methionine. Class II.3 includes other linear-non-pediocin-type enterocins such as enterocin B (Casaus et al., 1997). Class III regroups cyclic antibacterial peptides including enterocin AS-48 produced by *E. faecalis* S-48 (Maqueda et al., 2004). Finally, Class IV includes enterolysin A produced by *E. faecalis* (Nilsen et al., 2003). Recently, a glycosylated bacteriocin, enterocin F4-9, from *E. faecium* has been described by Maky et al. (2015). Only four bacteriocins produced by *E. durans* have been purified so far namely: durancin TW-49M a class II non-pediocin-like bacteriocin homologous to enterocin B (Hu et al., 2008), durancin GL produced by *E. durans* 41D and belonging to class II (Du et al., 2012), durancin L28-1A (Yanagida et al., 2005), and peptides A5-11A and A5-11B belonging to class II of bacteriocins with a high degree of similarity to enterocins L50A and L50B (Cintas et al., 2000; Batdorj et al., 2006). A large list of enterococci bacteriocins with their structure and spectrum of action is also available at BACTIBASE database (Hammami et al., 2010) (http://bactibase.hammamilab.org/Producer/Enterococcus).

### Wide-spectrum activity

Enterocins display a board spectrum of activity, they inhibit not only closely related species but also Gram-positive pathogens in particular, the genus *Listeria* (Khan et al., 2010). This attribute has been observed for example, in enterocin E-760 (Line et al., 2008), enterocin P (Cintas et al., 1997), enterocin LR/6 (Kumar and Srivastava, 2010), and Enterocin AS-48 (Cobo Molinos et al., 2008). Besides, enterocins DD28 and DD93 have been reported to be active against MRSA and prevented film formation (Al Atya et al., 2016). Similarly, strains *E. durans* 61A (Hanchi et al., 2016), *E. mundtii* ST15 (De Kwaadsteniet et al., 2005), and ST4SA (Dicks et al., 2010) have showed a broad spectrum of activity including Gram-negative bacteria, an unusual property of bacteriocins produced by LABs. Therefore, bacteriocin-producing bacteria could be used to get rid of Gram-negative pathogens in mammalian GITs (Kommineni et al., 2015). Indeed, bacteriocin production was shown to augment niche competition by enterococci in the mammalian GIT. Besides, some enterocins were shown to inhibit the growth of some mold spores such as durancin (Belguesmia et al., 2013). Nevertheless, a comprehensive assessment of the antifungal potential of enterocins is still lacking. Many enterocins have been reported to inhibit spore-forming bacteria, reviewed in Egan et al. (2016). Enterocin DD14 was reported being active against *Clostridium* (Caly et al., 2017), while durancin TW-49M (Hu et al., 2008) and Enterocin NKR-5-3B (Himeno et al., 2015) were potent against *Bacillus circulans*. Similarly, enterocin AS-48 displayed significant activity against endospores of *Alicyclobacillus acidoterrestris* and *Bacillus licheniformis* when combined with thermal treatment (Grande Burgos et al., 2014). Finally, bacteriocin ST5Ha produced by *E. faecium* ST5Ha (Todorov et al., 2010) and enterocin CRL35 produced by *E. mundtii* CRL35 (Wachsman et al., 2003) exhibit antiviral activities against herpes simplex viruses HSV-1 and HSV-2. Likewise, *E. mundtii* ST4V, isolated from soya beans, was also shown to produce a 3950 Da peptide ST4V with broad spectrum of inhibition against several bacteria and viruses including HSV-1, HSV-2, polio virus and measles virus (Todorov et al., 2005).

### Multi-bacteriocin producers

Enterococci are characteristically tolerant to extreme pHs, temperatures, and high salt concentration (Fisher and Phillips, 2009). Moreover, some enterococci strains harbor simultaneously many bacteriocin-related genes, which provide them a competitive advantage toward other microbial species in ecological niches (Vandera et al., 2017). These features are useful in food applications against spoilage and pathogenic organism contamination (Henning et al., 2015). Cintas and colleagues have reported that *E. faecium* L50, a producer of enterocin L50A and L50B, is able to produce two other additional bacteriocins (enterocin P and enterocin Q) at 37° and 47°C, respectively (Cintas et al., 2000). Likewise, *E. durans* 61A was recently shown to produce simultaneously formylated and nonformylated forms of enterocins L50A and L50B as well as durancin 61A, a new glycosylated 5217 Da peptide (Hanchi et al., 2016). Several other multi-bacteriocinogenic strains have been reported such as *E. faecium* NKR-5-3 (Perez et al., 2012), *E. faecium* WHE 81 (Izquierdo et al., 2009), *E. faecium* LM-2 (Liu G. et al., 2011) and *E. faecium* MMRA (Rehaiem et al., 2014).

## Current applications of enterococci and their bacteriocins

**Figure 2:**
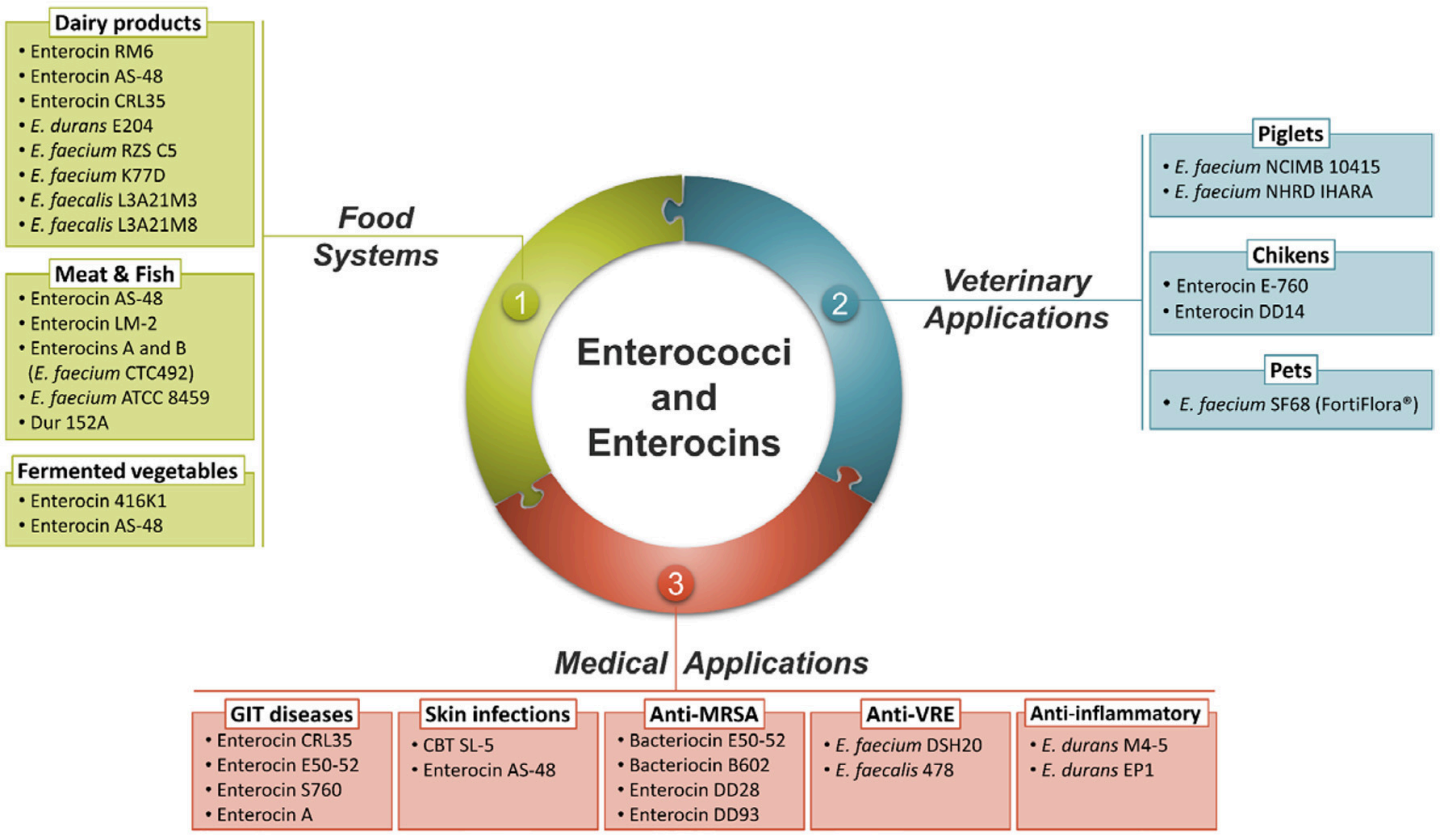
Summary of the main applications of enterococci strains and their bacteriocins.

As a normal inhabitant of the gut, *Enterococcus* strains are able to survive, compete and adhere to host cells in the GIT, important feature for a successful use as probiotics (Laukova et al., 2017). The genus *Enterococcus* also includes a wide range of strains suitable as starter cultures where they play a positive role in the development of the typical organoleptic characteristics of various fermented foods, including meat, dairy and vegetable products (Franz et al., 2011). Furthermore, the killing ability of bacteriocins produced by *Enterococcus* strains is considered a successful strategy for maintaining population and reducing the numbers of competitors (Yang et al., 2014). Over recent years, purified and identified enterococci bacteriocins were added to foods in the form of concentrated preparations or produced *in situ* by bacteriocinogenic starter, adjunct or protective cultures (Arqués et al., 2015). These bacteriocins are being considered as promising drug candidates for replacing antibiotics to treat multiple drugs resistant pathogens and maintain human health (Hammami et al., 2013). Bacteriocins demonstrated additive or synergistic effects in combination with other antimicrobial agents providing novel opportunities for more effective control of pathogens in human and veterinary medicine (Hammami et al., 2013). The current applications of *Enterococcus* strains and their bacteriocins as probiotics, in food, in veterinary and medical fields are summarized in Figure 2.

### *Enterococci* and their bacteriocins in food

The application of enterococci in food fermentation process is still debatable. Indeed, their presence in food is considered a sign of fecal contamination, but more recently they have been accepted as part of the normal microbiota and are commonly used in food industry (Franz et al., 2011). The genus *Enterococcus* is allocated into risk group 2, which includes microorganisms harboring virulence factors (EC, 2000). Thus, they can act as a reservoir for dissemination of antibiotic resistance and virulence genes through the food chain (Jahan et al., 2015). Horizontal gene transfer has been shown to be a mechanism of transmission between enterococci and other genera. *in vitro* studies have demonstrated that transfer between *Enterococcus* and *S. aureus* could occur (de Niederhausern et al., 2011). Nevertheless, horizontal gene transfer of the *van* operon between *Enterococcus* spp. and other organisms appears to occur at a very low frequency (Faron et al., 2016). As indicated above (Legislation section and Figure 1), the susceptibility to ampicillin and virulence factors and markers *IS16, hylEfm*, and *esp* are considered relevant for the assessment of safety (EFSA, 2012a). Recommended methods for the safety assessment of *Enterococcus* are summarized in Table 1. Safety is investigated through microbiological (hemolysis, production of gelatinase, antibiogram determination), molecular tests (antibiotic resistance genes and virulence factors, genes involved in the formation of BA), and detection of toxin production (for Non-QPS Strains). Finally, an assessment of lack of infectivity by the candidate strain in immunocompromised animals would add a measure of confidence in the safety (FAO/WHO, 2002). The amplification of the IS*16* gene by PCR as described by Werner et al. (2011) is recommended, while the hybridization technique described by Rice et al. (2003) is used for detection of *esp* and *hyl*Efm. As an alternative method, hybridization to colony lysates or Southern blots can be used. Both analysis should contain positive *E. faecium* ATCC BAA-472 (TX16) or *E. faecium* DSMZ 25390) and negative control (*E. faecium* DSMZ 25389) strains (EFSA, 2012a). Beside genotypic tests, phenotypic assessment is also needed. For instance, the determination of antibiotic's (mainly ampicillin) minimum inhibitory concentration (MIC) should be performed according to internationally recognized standards such as European Union Committee on Antimicrobial Susceptibility Testing (EUCAST), the Clinical and Laboratory Standard Institute (CLSI), ISO standard or similar. Currently, an ISO standard is available for bifidobacteria and non-enterococcal lactic acid bacteria. Strains with a MIC cut-off value above 2 mg/L are not considered safe and will not be authorized. The absence of hemolysis can be easily screened by plating on blood agar, while cytotoxicity could be assessed on Vero cells (Laulund et al., 2017). Thus, it has been suggested that application of enterococci in food could be admitted on the basis of careful selection and case-by-case studies (Giraffa, 2002; Foulquié Moreno et al., 2006).

**Table 1:** Recommended methods for the safety assessment of *Enterococcus* (non-QPS species).

**Tests**	**Methods**	**References**
**ANTIBIOTIC SUSCEPTIBILITY TESTS**		
Phenotypic antibiotic susceptibility: Ampicillin	Minimal inhibitory concentrations MICs (mg/L or μg/mL; Susceptibility Testing: EUCAST/ CLSI, ISO standard)	EFSA, 2012a
**OTHER CONSIDERATIONS**		
**Susceptibility to clinically relevant antibiotics** (vancomycin, gentamicin, kanamycin, streptomycin, erythromycin, clindamycin, tetracycline, chloramphenicol)	Minimal inhibitory concentrations (MICs) (mg/L or μg/mL; Susceptibility Testing: EUCAST/ CLSI, ISO standard)	EFSA, 2012b; Laulund et al., 2017
**DETECTION OF VIRULENCE MARKERS ASSOCIATED WITH THE HOSPITAL STRAINS**		
*IS16*	PCR	Werner et al., 2011
*esp*	Hybridisation techniques	Hendrickx et al., 2007
*hylEfm*	PCR	Rice et al., 2003
	*Alternative methods:* -Hybridisation to colony lysates -Southern blots	Singh et al., 1998
**OTHER CONSIDERATIONS**		
*Genotypic assessment	- Multilocus sequence typing (MLST) - DNA fingerprint - PCR	
**Vancomycin operons** (*vanA, vanB, vanC, vanD, vanE, vanG, vanM, vanL, vanN*)		Teo et al., 2011
**Surface adhesin genes** (*efaA_*fs*_, efaA_*fm*_*)		Eaton and Gasson, 2001
**Cytolysin genes** (*cylL_*L*_, cylL_*s*_, cylM, cylB, cylA*)		
**Aggregation protein gene** (*agg*)		
**Extracellular metalloendopeptidase** *gelE*		Nakayama et al., 2002
*Phenotypic assessment		
**Hemolytic Activity**	Hemolytic activity assay on 5% sheep or horse blood Columbia agar plates	Semedo et al., 2003
**Gelatin hydrolysis**	Assay for gelatinase activity on Todd-Hewitt (TH) agar plates containing 3% gelatin	Qin et al., 2000
**BIOGENIC AMINES DETECTION**		
- Histamine - Putrescine - Phenylethylamine - Cadaverine	- High pressure liquid chromatography HPLC	EFSA Panel on Biological Hazards, 2011
	*Alternative methods* (for Histamine) - Fluorometric methods - Immunoassays	FDA, 2011
	- Flow injection analysis	Hungerford et al., 2001
	- Colorimetric method	Patange et al., 2005
Detection of amino acid decarboxylase-positive microorganisms	- Quantitative real-time PCR histamine-producing LAB - *in vitro* detection method (Enzymatic or chemical analysis)	Landete et al., 2007; EFSA Panel on Biological Hazards, 2011; FDA, 2011
**TOXIN PRODUCTION**		
**- Cytotoxic potential**	Vero cell cytotoxicity test	Laulund et al., 2017
*Full genome (When available)*	NGS	EFSA, 2012a

The antimicrobial activity of bacteriocins against foodborne pathogens and spoilage bacteria has attracted considerable attention for their application in food preservation (Barbosa et al., 2017; Pisoschi et al., 2018). In addition, the use of bacteriocins can help reduce the use of chemical preservatives and/or intensity of heat and other physical treatments (Yang et al., 2014). In recent years, considerable efforts have been made to develop applications of bacteriocins and bacteriocinogenic strains in many food products. *E. faecium* and *E. faecalis* are bacteriocins producing species predominant in food (Cotter et al., 2005; Javed et al., 2011). Their bacteriocins can prevent the growth of several other bacteria such as *L. monocytogenes, Staphylococcus aureus* and *Vibrio cholerae*.

#### Dairy products

Enterococci among other LABs present in raw milk can act as natural starters. They also are able to survive during milk refrigeration and pasteurization temperatures due to their psychrotrophic nature, heat resistance and adaptability to different substrates and growth conditions (Bhardwaj et al., 2008). Enterococci are important in dairy industry and commonly present as non-starter LABs in cheese in Southern Europe. They are important in the maturation of different cheese varieties, probably due to their proteolytic or lipolytic activity, their ability to ferment citrate to produce diacetyl and other volatile compounds that contribute to the flavor or to provide a characteristic flavor and taste (Foulquié Moreno et al., 2006). For instance, *E. durans* and *E. faecalis* have been reported in raw and fermented milk and cheese products (Hanchi et al., 2014). Indeed, different species of enterococci are commonly found in cheese including *E. faecium, E. faecalis, E. durans*, and in a lesser extent *E. casseliflavus* (Ogier and Serror, 2008). Those cheeses are made from raw milk of goats, sheeps or cows. The UK Advisory Committee on Novel Foods has previously accepted the use of *E. faecium* K77D as a starter strain in fermented dairy products (ACNFP, 1996). In addition, several studies have been devoted to the detection, purification and characterization of the enterocins and their application as dairy food preservative. For instance, *E. faecalis* strains OSY-RM6, L3A21M3, and L3A21M8 produce thermolabile enterocins and *E. faecium* RZS C5 produces bacteriocins with activity against foodborne pathogens in milk and cheese (Leroy et al., 2002; Huang et al., 2013). More recently, a bacteriocin-like substance produced by *E. durans* E204 was proven to inhibit the growth of *L. monocytogenes* in cheese (Khay et al., 2014). Likewise, application of enterocins AS-48 and CRL35 inhibited spoilage and pathogenic microorganisms such as *S. aureus, L. monocytogenes* in different dairy products (Nuñez et al., 1997; Farias et al., 1999; Rodríguez et al., 2001; Arqués et al., 2015).

#### Fermented vegetables

The source of enterococci in-plant is not clearly defined, whether endogenous or an outcome of environmental contamination. The plant-associated enterococci species mainly include *E. faecium, E. mundtii, E. casseliflavus, E. faecalis*, and *E. sulfurous* (Müller et al., 2001). Enterococci were found in fermented green olives and are likely to be involved in the fermentation process of olives (de Castro et al., 2002; De Bellis et al., 2010; Corsetti et al., 2012; Lucena-Padrós et al., 2014). Those members are well adapted for initial pH value ?(9.0) and the salt concentration of the brine used in processing. This finding provides the rationale for using *E. faecium* and *E. casseliflavus* in the industrial fermentation of green olives (Corsetti et al., 2012). Some enterococci are also found as well in raw materials used in beer manufacture (Basanta et al., 2008). In addition, they are usually associated with sorghum (Madoroba et al., 2011) and soybean fermentations (De Kwaadsteniet et al., 2005; Kinouchi et al., 2012). Besides, *Enterococcus* bacteriocins are used as bioprotective agents in many vegetable food matrices such as fruit juices and ready-to-eat, fermented, and non-fermented vegetables (Settanni and Corsetti, 2008). For example, enterocin AS-48 is used in a wide range of vegetable products including cider, sliced fruits, vegetable juices, and canned vegetables (Grande Burgos et al., 2014). Use of enterocin AS-48 is considered effective in the inactivation of *Salmonella enterica* in contaminated fruit juice (Martinez Viedma et al., 2008), and *L. monocytogenes* and spore-forming microorganisms in raw and canned vegetables (Lucas et al., 2006; Cobo Molinos et al., 2008). Similarly, enterocins CCM4231 and EJ97 were used in soymilk and zucchini purée for controlling *Listeria* and *Bacillus*, respectively (Lauková and Czikková, 1999; García et al., 2004).

#### Meat products

Enterococci are normal constituents of the natural microbiota of many fermented-meat products, but can also be found in raw meat (Garriga and Aymerich, 2014), with *E. faecium* and *E. faecalis* being the predominant species, followed by *E. hirae* and *E. durans* (Franz et al., 2011). *E. faecium* and *E. mundtii* are reported in fish and fermented seafood (Ishibashi et al., 2012), and chicken and fermented meat products such as sausages (Hugas et al., 2003; Barbosa et al., 2014). Their presence could contribute in the development of sensory properties of fermented meat products particularly in sausage (Hugas et al., 2003). Other important functional properties including metmyoglobin-reducing activity, ability to degrade anti-nutritive factors like stachyose and raffinose, and production of bile salt hydrolase have also been described for meat enterococci (Omar et al., 2004). In addition to desired fermentation properties, the bacteriocin-producing *Enterococcus* strains are used as protective cultures used in ready to eat meats and other products (Foulquié Moreno et al., 2006). In fact, the processing conditions, the curing additives, and the presence of lactic starter cultures are effective for pathogen control but not sufficient to prevent the survival of pathogens like *E. coli* O157:H7, *L. monocytogenes* and *Salmonella* during the manufacturing process (Castellano et al., 2017). In this regard, *E. faecium* RZS C13 and CCM 4231, and *E. casseliflavus* IM416K1 were shown competitive and strongly inhibited the growth of *Listeria* spp. when used as starter cultures for the production of fermented sausage (Callewaert et al., 2000; Sabia et al., 2003). Nevertheless, *in situ* bacteriocin production rely on many factors such as the strain ability for food colonization, thermal treatment, refrigeration, and presence of other preservative agents such as nitrites, sodium chloride, organic acids and other ingredients (Castellano et al., 2017). For example, *E. faecium* CTC492, an enterocins A and B producer, did not significantly reduce *Listeria* counts in fermented sausages, which was attributed to a higher inhibition of the producer strain by refrigeration temperatures and sausage ingredients (Aymerich et al., 2000). In contrast, bacteriocins are heat resistant, they can be added in, or on, foods that may be heated or cooked (Vijayakumar and Muriana, 2017). Consequently, several successful applications of enterococci bacteriocins have been reported, mainly concerning their effectiveness in eliminating *L. monocytogenes, Salmonella, Clostridium*, and other spoilage lactic bacteria. For instance, significant reduction of listerial cells in ham was observed after addition of partially purified bacteriocins from *E. durans* 152 (an enterocin L50A derivative and enterocin L50B), with anti-listerial protection lasted for at least 30 days at 15°C (Du et al., 2017). Unlikely regrowth of the listerial cell survivors was observed after 1 h of challenge with enterocin CRL35 (Vignolo et al., 2000). Conversely, the application of enterocin 416K1 and semi-purified enterocins A and B on low-density polyethylene and alginate/zein/PVA films, respectively, reduced *L. monocytogenes* counts by 5 logs in contaminated frankfurters and cooked ham at refrigeration temperature (Marcos et al., 2007; Iseppi et al., 2008). Likewise, enterocin AS-48 alone decreased *L. monocytogenes* count and significantly inhibited *Salmonella* when combined with high hydrostatic pressure at the end of ripening of low acid fermented sausage (Ananou et al., 2010). The use of bacteriocins combined with other hurdle technologies may represent a useful approach to enhance antimicrobial effectiveness. Indeed, Turgis et al. (2012) and Liu et al. (2012) reported an improved anti-listerial effect of bacteriocin MT 104 and partially purified enterocin LM-2 combined with γ-irradiation and high hydrostatic pressure, respectively, in sausage and cooked ham.

Due to the activities of meat microbiota, undesirable reactions can also take place, such as the formation of biogenic amines. The presence of biogenic amines in food constitutes a potential public health concern because of their physiological and toxicological effect. At high concentrations, they are known to cause food poisoning, due to their vasoactive properties (Bargossi et al., 2017). The most dangerous are histamine (produced from histidine) and tyramine (produced from tyrosine) and are regarded as undesirable compounds that may cause health disruptions to sensitive consumers (EFSA Panel on Biological Hazards, 2011). Besides food poisoning, these substances affect the freshness and other organoleptic properties of meat and meat products (Hugas et al., 2003). Biogenic amines are mainly accumulated in foods through microbial decarboxylation of certain amino acids (Gardini et al., 2016). *Enterococcus* species, together with other lactic acid bacteria, constitutes one of the microorganisms that can accumulate higher biogenic amines; they are indeed known as the most efficient tyramine producers in fermented foods (Ladero et al., 2012; Bargossi et al., 2017; Laukova et al., 2017). Therefore, control measures to prevent biogenic amine formation in foods or to reduce their levels once formed must be taken. Hydrostatic pressures, irradiation, controlled atmosphere packaging, represent an important technological tool for controlling biogenic amine production (Naila et al., 2010). In addition, modeling the microorganisms responsible for biogenic amine formation has been proposed to control biogenic amine accumulation (Gardini et al., 2008). However, these approaches may not be efficient. Another tool able to counteract biogenic amines accumulation in fermented meats is to use starter cultures free of any decarboxylating activity and able to inhibit decarboxylase-positive bacteria (Latorre-Moratalla et al., 2012; Gardini et al., 2016). It has been demonstrated that using bacteriocinogenic strains and/or bacteriocins could contribute to reduce the risks of survival and multiplication of amino-biogenic bacteria during ripening and storage of fermented foods (Joosten and Nunez, 1996; Tabanelli et al., 2014). For instance, Laukova et al. (2017) tested the sensitivity of biogenic amines-producing *Enterococcus* strains from poultry to seven partially purified enterocins and found that although these strains featured high biogenic amines production, they were sensitive to the tested enterocins. Earlier, the application of enterocin AS-48 reduced biogenic amines-forming LABs in sardine filets (Ananou et al., 2014). The levels of other biogenic amines such as cadaverine, putrescine, tyramine, and histamine were significantly reduced by several folds in the enterocin AS-48-treated samples after storage, reviewed in (Grande Burgos et al., 2014). Likewise, *E. faecium* MCL13 was found to produce an antibacterial compound with inhibitory activity against the tested histamine-producing bacteria and exhibited histamine-degradation ability in fermented fish (Lim, 2016). Furthermore, the production of biogenic amines during fermentation can be controlled by the use of amine oxidizing bacteria and enzymes (Naila et al., 2010). Only a single study reported this ability to degrade biogenic amines among *Enterococcus* (Guarcello et al., 2016).

### *Enterococci* as probiotics

Many studies have been conducted to evaluate the probiotic characteristics of *Enterococcus* strains, mainly *E. faecium*. Due to safety concerns, lack of safety information, and legislation, only a limited number are commercialized. *Enterococcus* has not yet obtained the status GRAS (Franz et al., 2011). However, some strains such as *E. faecium* M74 and *E. faecium* SF-68 are included as food supplements in several probiotic preparations, that have been proved to be effective and safe, such as Cernivet® and FortiFlora® (containing *E. faecium* SF68®, Cerbios-Pharma SA, Switzerland), and Symbioflor® 1 with *E. faecalis* (Symbiopharm, Herborn, Germany) (Serio et al., 2010). Enterococci probiotics can be used in treatment and/or prevention of certain human and animal diseases such as alleviation of irritable bowel syndrome symptoms and antibiotic-induced diarrhea and prevention of different functional and chronic intestinal diseases (Bybee et al., 2011). Moreover, some enterococci exhibit anticarcinogenic, hypocholesterolemic, as well as immune regulation effects. For instance, *E. durans* M4-5 has been found to generate butyrate, a short chain fatty acids (SCFAs) that induce significant anti-inflammatory effects and contribute to the integrity of the intestinal epithelium (Avram-Hananel et al., 2010). Similarly, administration of *E. faecium* M74® was associated with a reduction of the cholesterol concentration in serum (Hlivak et al., 2005). Furthermore, *E*. *durans* KLDS 6.0930 has been postulated as a probiotic candidate through lowering human serum cholesterol levels (Liu et al., 2016). *E. mundtii* ST4SA was recently presented as another potential probiotic strain (van Zyl et al., 2016). It generates antimicrobial peptides that have activity against a number of pathogens supporting its antimicrobial and probiotic roles (Ramiah et al., 2009). More recently, the strain *E. durans* LAB18s was recommended useful for use as a source of dietary selenium supplementation (Pieniz et al., 2014), while *E. faecium* LCW 44 and *E. durans* 6HL were shown highly potent against Gram-positive (Vimont et al., 2017) and Gram-negative bacteria (Nami et al., 2014; Pieniz et al., 2014; Vimont et al., 2017), respectively.

The application of probiotics in animal nutrition needs prior authorization (EFSA, 2012b; Figure 1). In feed regulation, probiotics are included in the group of feed additives for stabilizing the microbial communities of the digestive tract in both monogastric and ruminant animals (Anadon et al., 2006). For instance, The European Food Standards Agency (EFSA) authorized certain strains of enterococci for use as silage additive and dietary supplements (EFSA, 2012a). Strains *E. faecium* NCIMB 11181 and *E. faecium* DSM 7134 were approved as feed additives for calves and piglets by EFSA (EFSA, 2012b). The probiotics *E. faecium* SF68® (NCIMB 10415 Cerbios- Pharma SA, BARBENGO, Switzerland) and *E. faecalis* Symbioflor 1 (Symbiopharm, Herborn, Germany) are also used to prevent or treat diarrhea in pigs, poultry, livestock and pets (Bybee et al., 2011; Franz et al., 2011). Probiotics have been shown to have positive effects on the performance characteristics of the growth and health of farm animals. Feeding pigs with the probiotic *Enterococcus* was found to reduce intestinal pathogens (Liao and Nyachoti, 2017). Similarly, the probiotic *E. faecium* NCIMB 10415 has increased the capacity of absorption and secretion of mucous membranes in jejunum and improved the intestinal barrier integrity (Bednorz et al., 2013). Likewise, oral administration of *E. faecium* NHRD IHARA by post-weaning piglets has increased serum and fecal IgA levels and improved piglets growth (Sukegawa et al., 2014). In chickens*, E. faecium* was demonstrated to improve growth, intestinal morphology, and the caecal microbiota homeostasis (Cao et al., 2013). *E. faecium* was also reported to *improve* the metabolic efficiency and decrease inflammatory responses in broilers (Zheng et al., 2016). Additionally, enterocin E-760 (Line et al., 2008), and enterocin DD14 (Caly et al., 2017) were used to control *Campylobacter spp*. and *C. perfringens* infections and spread in chicken (Ščerbová and Lauková, 2016). Enterococci are also used as probiotics for dogs based on their tolerance to bile, adhesion activity, antimicrobial activity and their impact on high levels of serum cholesterol and alanine aminotransferase (Bybee et al., 2011). Numerous studies have shown the beneficial effects of enterococci in aquaculture. Several studies reported a wide spectrum of inhibition by *E. faecium* toward aquatic pathogens including *Yersinia ruckeri, Vibrio harveyi, Streptococcus agalactiae* and *Aeromonas veronii* (Swain et al., 2009; Satish Kumar et al., 2011). In addition, several trials have investigated the efficacy of *E. faecium* incorporated in feed to improve fish growth (Bogut et al., 2000; Chang and Liu, 2002) and stimulate immune response (Panigrahi et al., 2007; Román et al., 2015).

## Therapeutic potential of enterococcal bacteriocins

Bacteriocins are an interesting alternative to the use of antibiotics, which have created great public concerns due to the emergence of antimicrobial resistance. New compounds and therapeutic methods for treating infections caused by antibiotic-resistant pathogens and limiting their spread are urgently needed. Hence, there is a need to discover new antimicrobial agents and to develop innovative strategies to fight against those pathogens (Hammami et al., 2013).

### Treatment of gastro-intestinal diseases

Gastrointestinal diseases are usually associated with gut microbiota dysbiosis. Probiotics affect the functionality of the GIT by a variety of mechanisms such as interfering with the attachment of pathogens to adhesion sites, out-competing pathogens for nutrients, degradation or other alterations of toxin receptors, production of inhibitory substances (e.g., bacteriocins and/or organic acids) and stimulation of immunity/immunomodulation (Fliss et al., 2011). Species such as *H. pylori, C. difficile, L. monocytogenes*, and *Salmonella* are the main bacteria involved in severe gastric infections (Hammami et al., 2013). The broad inhibitory spectra of bacteriocins produced by *Enterococcus* make them appropriate candidates, alone or in combination with other antibiotics, to prevent or treat these infections. For example, administration of enterocin CRL35 alone showed significant activity against *L. monocytogenes*, while the genetically engineered hybrid peptide Ent35-MccV displayed a broad spectrum antimicrobial activity against enterohemorrhagic *E. coli* and *L. monocytogenes* clinical isolates (Acuña et al., 2012; Salvucci et al., 2012). Moreover, enterocin S760 and enterocin A have significantly reduced *Bacillus anthracis* and *Salmonella* infection in mice and quail, respectively (Lauková et al., 2003; Svetoch et al., 2011). Likewise, enterocin E50-52 has effectively been used to treat infection in birds (Svetoch et al., 2008). More recently, durancin 61 A has demonstrated a significant activity when combined with reuterin against *C. difficile* providing a possible therapeutic use for the treatment of gastrointestinal infections (Hanchi et al., 2017). Although immunomodulation is one of the main mechanisms of action of probiotic bacteria, very few studies have actually documented immunomodulatory properties of *Enterococcus*. For example, *E. faecium* L5 has increased the expression of IL-10 and decreased the IL-8 while *E. faecalis* CECT 7121 and *E. faecium* JWS 833 have enhanced the cytokine production (Tarasova et al., 2010; Choi et al., 2012; Molina et al., 2015). In a lesser extent, Kanda and collaborators have demonstrated that the administration of *E*. *durans* TN-3 suppressed the development of dextran sodium sulfate DSS colitis via the induction of IL-10 producing Treg cells (Kanda et al., 2016). Another recent study have reported that *E. durans* EP1 modulates gut microbiota by increasing *Faecalibacterium prausnitzii*, a functionally important bacterium in some cases of dysbiosis (Carasi et al., 2017).

### Treatment of skin infections

Enterocins are being extensively studied for use in treatment of acne (Figure 2). For example, CBT SL-5 lotion prepared with bacteriocin produced by *E. faecalis* SL-5 has significantly reduced the inflammatory lesions caused by *Propionibacterium acne*s, suggesting a potential role in acne treatment as an alternative to antibiotics (Kang et al., 2009). A recent clinical trial demonstrated the leishmanicidal effect of enterocin AS-48, which was lethal on both axenic promastigotes and amastigotes of *Leishmania* at low micromolar concentrations with scarce toxicity on the host cell (Abengózar et al., 2017). Previously, a patent was issued for the use of AS-48 combined with lysozyme for applications against acne and other skin bacterial infections targeting *P. acnes* and *S. aureus* (Maqueda Abreu et al., 2014).

### Treatment of infections caused by antibiotic-resistant bacteria

Nosocomial infections involving drug-resistant bacteria are a major concern in hospitals. They contribute to increased morbidity, length of stay and the cost of care. The main multi-resistant organisms include methicillin-resistant *S. aureus* (MRSA), vancomycin *resistant Enterococci* (VRE) and *Enterobacteriaceae* members that generate extended-spectrum beta-lactamase (ESBL) (Lebreton et al., 2013). Because of their activity against clinically important strains and synergistic activity with other bacteriocins and antibiotics, enterocins could be used in the treatment of nosocomial infections caused by these resistant bacteria (Hammami et al., 2013).

#### Treatment of methicillin-resistant staphylococcus aureus (MRSA)

Methicillin-resistant *S. aureus* (MRSA) is usually resistant to oxacillin, cloxacillin, and other semi-synthetic antibiotics related to penicillin. They may also be resistant to tetracycline, clindamycin, cephalosporins, macrolides, quinolones and other antibiotics. In community, most MRSA infections are skin and soft tissue infections. In medical establishment, MRSA causes bacteraemia, septicemia, endocarditis, pneumonia and surgical site infections (Lee et al., 2016). The efficacy of vancomycin, the traditional antibiotic chosen to treat these infections, has declined with the emergence of resistant strains (Fair and Tor, 2014). Alternatively, bacteriocins E50-52 and B602 have been proven effective against antibiotic-resistant strains in nosocomial infections (Svetoch et al., 2009). Additionally, enterocins DD28 and DD93 were identified as anti-MRSA agents (Al Atya et al., 2016). Similarly, durancin 61A alone or in combination with vancomycin was shown effective against clinical MRSA (Hanchi et al., 2017), which may therefore provide a possible therapeutic option for the treatment of MRSA infections.

#### Treatment of vancomycin-resistant enterococci (VRE)

The spread of VRE may lead to clinical isolates resistant to all antibiotics because enterococci have become important nosocomial pathogens and a reservoir for resistance genes (Fair and Tor, 2014). Interestingly, two peptides produced by *E. faecium* DSH20 (35 kDa) and *E. faecalis* 478 have shown potent activities against VRE (Shokri et al., 2014; Phumisantiphong et al., 2017) which may provide an alternative therapy for drug-resistant strains. Similarly, durancin 61A was shown effective against VRE clinical isolates (Hanchi et al., 2017).

### Anti-inflammatory activity

*E. durans* M4-5 was found to produce butyrate, a metabolic product that induces significant anti-inflammatory effects and contributes to intestine epithelial integrity. This novel anti-inflammatory bacterium may be preferentially useful as a prophylactic treatment to avoid inflammatory bowel disease (Avram-Hananel et al., 2010). More recently, administration of *E. durans* EP1 was found to increase the amount of *Faecalibacterium prausnitzii*, a butyrate-producing bacteria, which is known for its anti-inflammatory effects (Carasi et al., 2017).

## Conclusions and perspectives

A growing number of studies have illustrated the beneficial effects of probiotics on health including the elimination of pathogens. Until recently, the most commonly used probiotic strains are related to *Bifidobacterium, Lactobacillus*, and *Lactococcus* species. In order to identify new members, other microorganisms with probiotic potential should be evaluated for probiotic candidacy. Among these candidates, *Enterococcus* spp. are prominent. However, for safety reasons, the application of enterococci as probiotic or feed additive has not been sufficiently exploited despite their significant antibacterial activity and probiotic potential (Franz et al., 2011). This genus has bad reputation due to members associated with severe health-care associated infections such as vancomycin-resistant enterococci (VRE). There is a major concern about use of *enterococci* strains in food supplements, which could lead to the spread of multi-resistance and virulence genes (Jahan et al., 2015). In an even more alarming development, transfer of vancomycin resistance from enterococci to methicillin-resistant strains of *S. aureus* has been reported in more than one study (de Niederhausern et al., 2011). Thus, strains carrying acquired resistance should not be intentionally introduced to the food and feed chain. In the case of feed and novel foods, a pre-market safety assessment is required where the safety of the candidate strains is assessed at species-level. In all cases, EFSA guidance documents on strain safety should be followed (EFSA, 2012a). Recent advances in molecular biology have demonstrated that enterococcal food strains are safe and can be differentiated from the nosocomial strains carrying virulence and antimicrobial resistance genes (Montealegre et al., 2016). This will enable possible improvement of the safety assessment of enterococci used in food and feed.

The genus *Enterococcus* is a member of LABs and constitutes a part of human-associated microbiota including in mouth, skin, and GIT. Some *Enterococcus* strains have many interesting properties such as multi-bacteriocin production and viability in different matrices including food and GIT, which highlight their potential use as natural preservatives in food, as probiotics, or as viable alternatives to antibiotics. In addition, enterococci bacteriocins are recognized for their wide spectrum antimicrobial activity including Gram-positive foodborne pathogens, such as biogenic amines producing bacteria (Laukova et al., 2017), *L. monocytogenes* and Gram-negative bacteria. Moreover, some bacteriocins possess antifungal and/or antiviral activity and can also inhibit sporulating bacteria such as *C. botulinum* and *B. cereus* and in some case they may inhibit endospores (Grande Burgos et al., 2014). These features provide the rationale to nominate bacteriocinogenic *Enterococcus* strains as important candidates for food, human and animal health applications.

Furthermore, bacteriocin production is an important element in competition among bacteria. Bacteriocin-producing probiotics could compete with intestinal pathogens for colonization or modulate the microbiota homeostasis. In this context, it has been reported that bacteriocins can be produced in the gut by probiotic bacteria where it can modulate gut microbiota to reduce gastrointestinal diseases (Salvucci et al., 2012; Cotter et al., 2013). However, the mechanisms by which probiotics attenuate gastrointestinal infections need to be evaluated in order to determine their efficacy more accurately. Bacteriocins are target specific, safe, can synergize with antibiotics, and are heat stable, interesting features for the development of drug candidates to the treatment of antibiotic-resistant bacterial infections. Considering the emergence of resistance, it is believed that multiple bacteriocin productions can help the producer strain to abolish resistance problem of some target strains (Perez et al., 2012). The use of bacteriocins in combination with other antimicrobials (Hanchi et al., 2017), or developing new products via peptide engineering is also a therapeutic option that is increasingly efficacious as resistance spreads (Cotter et al., 2013). In general, the mainstream use of bacteriocin therapies will need careful and controlled implementation to limit possible resistance development.

Finally, probiotics have been defined as live microorganisms which, when administered in adequate amounts, confer a health benefit to the host. This definition suggests that safety and efficacy of probiotics have to be demonstrated for each strain and each product. As probiotic properties have been shown to be strain specific, accurate identification of candidate strains is also very important. In spite of their pathogenic potential, commensal enterococci generally display low levels of virulence, as evidenced by their presence as natural colonizers of the GIT of humans and most animals and by the fact that they have been used safely for decades as probiotics in humans and farm animals (Arias and Murray, 2012). Therefore, their application as promising probiotics in food and feed industry needs implementation of appropriate guidance and relevant legislation of valid scientific methods to distinguish between pathogenic and non-pathogenic stains, and to prevent horizontal transfer of pathogenic genes. Despite the several hurdles that must be overcome for the exploitation of enterococci and their bacteriocins in food systems, as probiotics and in drug discovery, the innovations and developments discussed in this review offer a taste of future trends in food, veterinary and pharmaceutical applications of these intriguing microbes.

## Author contributions

HH and RH designed the manuscript. HH wrote the manuscript. WM, KS, and RH critically evaluated the manuscript.

### Conflict of interest statement

The authors declare that the research was conducted in the absence of any commercial or financial relationships that could be construed as a potential conflict of interest.
